# kmlShape: An Efficient Method to Cluster Longitudinal Data (Time-Series) According to Their Shapes

**DOI:** 10.1371/journal.pone.0150738

**Published:** 2016-06-03

**Authors:** Christophe Genolini, René Ecochard, Mamoun Benghezal, Tarak Driss, Sandrine Andrieu, Fabien Subtil

**Affiliations:** 1 Inserm UMR 1027, University of Toulouse III, Toulouse, France; 2 CeRSM (EA 2931), UFR STAPS, University Paris Ouest-Nanterre-La Défense, Nanterre, France; 3 Service de Biostatistique, Université Lyon 1, Villeurbanne, France; 4 CNRS, UMR5558, Equipe Biotatistique-Santé, Laboratoire de Biométrie et Biologie Evolutive, Villeurbanne, France; 5 Department of Epidemiology and Public Health, CHU Toulouse, Toulouse, France; University of Connecticut, UNITED STATES

## Abstract

**Background:**

Longitudinal data are data in which each variable is measured repeatedly over time. One possibility for the analysis of such data is to cluster them. The majority of clustering methods group together individual that have close trajectories at given time points. These methods group trajectories that are locally close but not necessarily those that have similar shapes. However, in several circumstances, the progress of a phenomenon may be more important than the moment at which it occurs. One would thus like to achieve a partitioning where each group gathers individuals whose trajectories have similar shapes whatever the time lag between them.

**Method:**

In this article, we present a longitudinal data partitioning algorithm based on the shapes of the trajectories rather than on classical distances. Because this algorithm is time consuming, we propose as well two data simplification procedures that make it applicable to high dimensional datasets.

**Results:**

In an application to Alzheimer disease, this algorithm revealed a “rapid decline” patient group that was not found by the classical methods. In another application to the feminine menstrual cycle, the algorithm showed, contrarily to the current literature, that the luteinizing hormone presents two peaks in an important proportion of women (22%).

## 1 Introduction

### 1.1 Clustering longitudinal data

Longitudinal data are data in which each variable is measured repeatedly over time. One way of analyzing this type of data is to cluster them; i.e., divide the population into homogeneous subgroups. For this, different methods were proposed among which variants of k-means [1–6] and various model-based classification methods relying on mixture models [7–11]. The pros and cons of these approaches are regularly discussed [12, 13] though there are no current recommendations on which method to prefer in a specific context.

**Fig 1:**
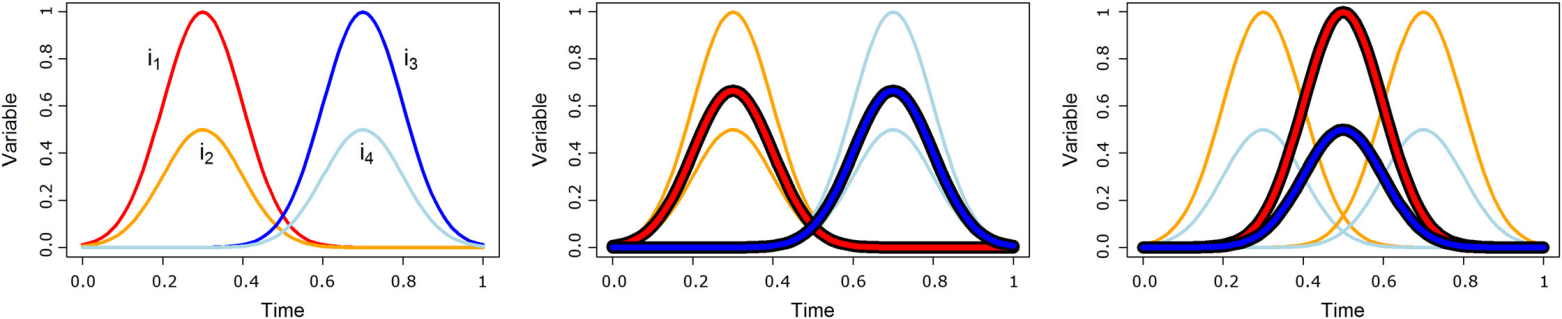
Cluster longitudinal data according to their shapes. (a) four trajectories. (b) With classical techniques, trajectories *i*_1_ and *i*_2_ (in orange) belong to the same cluster A while *i*_3_ and *i*_4_ (light blue) belong to another cluster B. The mean of cluster A is in red; that of cluster B is in deep blue. (c) Using “shape-respecting clustering”, *i*_1_ and *i*_3_ (in orange) belong to cluster A while *i*_2_ and *i*_4_ (light blue) belong to cluster B. The shape-respecting mean is in red for cluster A and in deep blue for cluster B.

The general idea behind partitioning is to group similar individuals within the same cluster. Different approaches to the concept of “similarity” are possible. They may be based on the concept of distance, resemblance, or likelihood. In the majority of the currently available approaches, two individuals are considered similar when they have close trajectories at each time point. This approach takes into account local similarities but not necessarily the general shapes of the trajectories. In particular, two identical trajectories but shifted in time are considered different and may be potentially assigned to distinct clusters. The immediate consequence is that the mean of the group does not inform on the shapes whereas, in a number of cases, the progress of a phenomenon may be more important than the moment at which it occurs. In such circumstances, one would prefer a partitioning that groups individuals whose trajectories have similar shapes whatever the shift in time. An example of this is shown Fig 1. With classical techniques, trajectories *i*_1_ and *i*_2_ (in orange) belong to the same cluster A while *i*_3_ and *i*_4_ (light blue) belong to another cluster B. The mean of cluster A is in red; that of cluster B is in deep blue. Using “shape-respecting clustering”, *i*_1_ and *i*_3_ (in orange) belong to cluster A while *i*_2_ and *i*_4_ (light blue) belong to cluster B. The shape-respecting mean is in red for cluster A and in deep blue for cluster B.

### 1.2 Shape respecting tools

The problems of trajectories with similar shapes were mainly addressed in two ways: i) distances and ii) means.

Intuitively, a distance is a function that takes two individuals and returns a number. The number should have a low value when the two individuals are close and a high value when the two individuals are distant from each other. A shape-respecting distance is a distance that takes a small value when individuals have trajectories with similar shapes but a big value otherwise.

Several shape-respecting distances have been proposed in the literature. The most studied are the Fréchet distance [14] and the Dynamic Time Warping [15–18] but there are many other alternatives like HCCA [19] or EDR (Edit Distance is Real sequence) [20] or longest common subsequence [21].

The problem of the mean respecting the form of trajectories is more complex. Many solutions exist. Curve alignment consists in deforming the trajectories so as to align some specific points (minimums, maximums, inflexion points) [22–27]. In a second step, the deformed curves are modeled according to mixture modeling. Or a simple Euclidean means is computed on the deformed trajectory.

More recently, for a higher efficiency, a number of authors [28, 29] have chosen to partition and align jointly. Currently, they are only few articles on method performance comparisons [27, 30]; most articles tend to show that one of the most efficient methods is fdakma [31].

Unfortunately, most methods suffer from various weaknesses; mainly, they are efficient only in populations with well-separated clusters and limited shifts.

### 1.3 Clustering according to shape

Using k-means with a classic distance does not allow solving the similar-shape clustering problem. But using a shape distance does not allow solving it either. Indeed, the use of the shape distance will form correctly the clusters by grouping individuals whose trajectories have similar shapes, but the mean trajectory of each cluster will not necessarily be representative of the group. Thus, the following iterations are affected.

**Fig 2:**
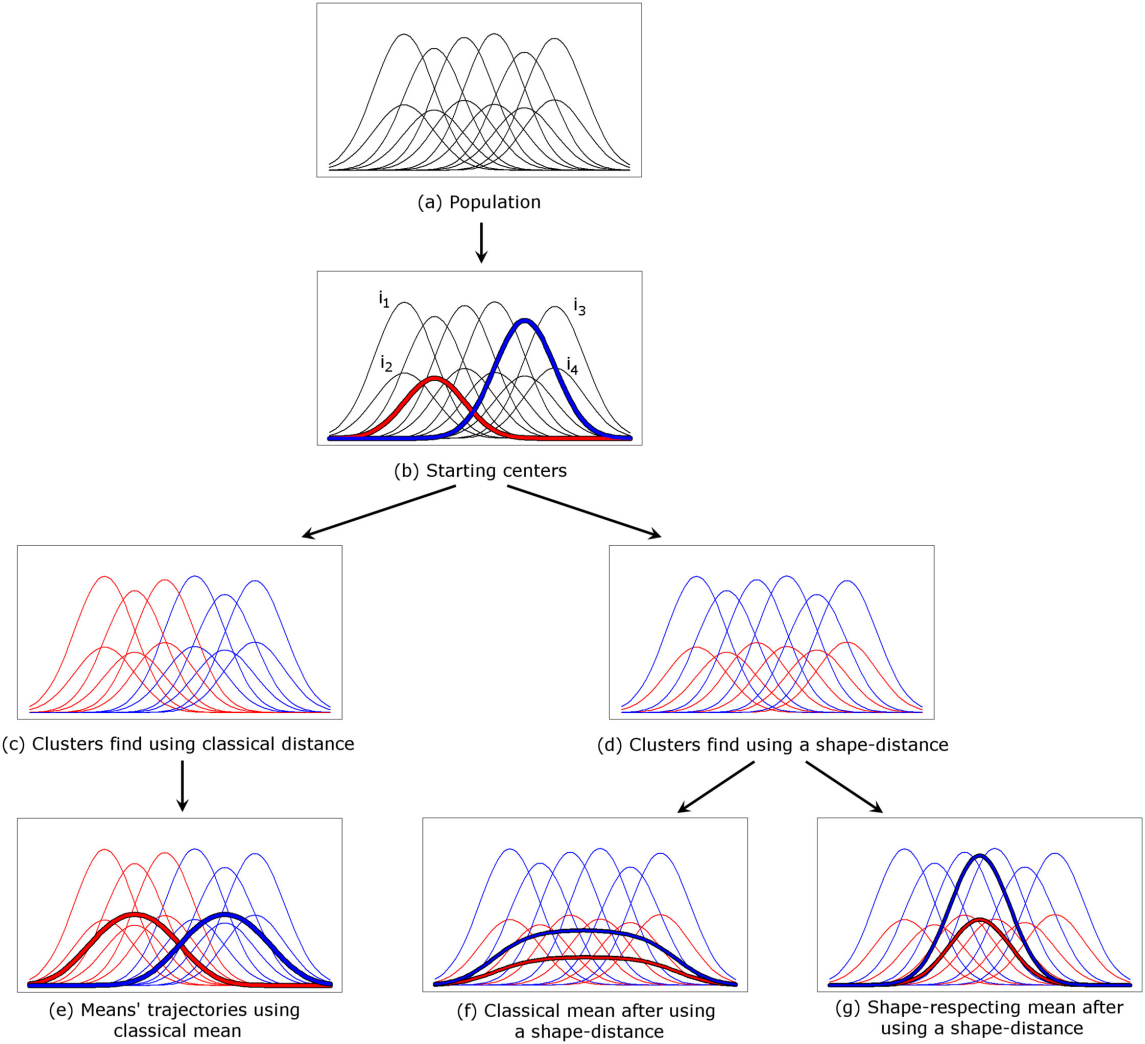
The impact of using the classical distance, the classical mean, the Fr´echet distance and the Fr´echet mean.

Fig 2 gives an illustration of the impact of the methods on the partitioning process. The population is shown Fig 2.a. It is a mixture of two groups of trajectories: one whose tops are high (between 0.75 and 0.85) and the other whose tops are lower (between 0.35 and 0.45). The objective of the algorithm is to identify the two groups. During the initialization phase, two individuals are randomly chosen (red and blue, Fig 2.b). The expectation phase assigns each individual to the closest cluster. By using the Euclidean distance, both individuals *i*_1_ and *i*_2_ are close to the red individual and will be classified in the red group whereas *i*_3_ and *i*_4_ will be classified in the blue group. This method leads to the partition presented Fig 2.c, then to the mean trajectories shown Fig 2.e. This partition does not find the two groups that constituted the initial population. Using a shape-respecting distance, individuals *i*_1_ and *i*_3_ are close to the individual in blue and will be classified in the blue group whereas *i*_2_ and *i*_4_ will be classified in the red group. This method leads to the partition presented Fig 2.d. Now, using a conventional way to compute the mean leads to find the mean trajectories presented Fig 2.f. The groups identified this way are correct, but the mean trajectories are not representative. The use of a shape-respecting mean leads to find the mean trajectories shown in Fig 2.g. The groups are correct and the mean trajectories are representative of the groups.

In the present article, we introduce kmlShape, a new partitioning method that clusters trajectories according to their shapes.

This method is based on a variation of the k-means algorithms in which we use a “shape-respecting distance” and a “shape-respecting mean”. Regarding the shape-respecting distance, we define a new method, the “generalized distance of Fréchet” which is a generalization of both the Fréchet distance and the Dynamic Time Warping. Regarding the shape-respecting mean, we define a new curve alignment solution. It is based on the construction of the Fréchet mean between two curves, then between n curves.

This method can be time consuming in case of large datasets. We introduce thus two methods that reduce the data size while keeping the essential information contained in the initial trajectories. The use of both data reduction and kmlShape yields a partitioning method that preserves the shapes of the trajectories and may be used with high-dimensional data.

The sections below are organized as follows: we present first the methods used to partition the trajectories according to their shapes. Next, the performances of the methods are evaluated with artificial and real data. Then we discuss the results, the quality of the partitioning on the artificial data, and the originality of the method with real data; i.e., the ability of the algorithm to reveal clusters that are undetectable with the classical methods.

## 2 Methods

### 2.1 General considerations

#### 2.1.1 Notation

Let us consider a set *S* of *n* subjects. For each subject *i*, an outcome variable *Y* is measured *t* times. For the sake of simplicity, we consider that all trajectories have the same number of measurements *t* though trajectories with different numbers of measurements do not add complexity to the algorithm. The time of the *j*^*th*^ measurement for subject *i* is noted *x*_*ij*_. The value of the *j*^*th*^ measurement for subject *i* is noted *y*_*ij*_. The sequence $Y_{i.}=((\begin{array}{c} x_{i1}\\ y_{i1} \end{array}),(\begin{array}{c} x_{i2}\\ y_{i2} \end{array}),...,(\begin{array}{c} x_{it}\\ y_{it} \end{array}))$ is called a trajectory.

#### 2.1.2 k-means using shape-respecting distances

k-means is a partitioning algorithm that belongs to Classification-Expectation-Maximization (CEM) methods [32]. This algorithm was first used with classical data [33, 34] but is now widely used with longitudinal data in various fields [5, 35–40]. The principle of k-means is to alternate two steps: i) an Expectation step that calculates the distances between the individual trajectories and the mean trajectories of each cluster; then each individual is assigned to the closest cluster; ii) a Maximization step that estimates the mean trajectory of each cluster. Before alternating these two steps, an initialization phase defines the “mean trajectories of each cluster” that will be used in the first maximization step. Various initialization methods are possible, as detailed in [41–44]. Here, we use the classical method that selects randomly *k* inidividuals and considers them as the *k* first clusters’ centers.

kmlShape is a new clustering algorithm that clusters trajectories according to their shape. It applies k-means within the context of a shape-respecting partitioning. As briefly reminded here, method k-means uses two tools: a distance and a mean. kmlShape requires both a distance and a mean that take the shapes into account. These tools (Fréchet distance and Fréchet means) are presented in the next section.

Overall, kmlShape is a variant of k-means using: i) the Fréchet distance to calculate the distances between individuals and cluster centers; ii) Fréchet mean to construct the centers of the clusters. The stopping condition is the stability of the algorithm: when the clusters are identical at step *s* and step *s* − 1, the algorithm is terminated (with a limitation of the number of iterations to avoid very long times before convergence). The pseudo code of the algorithm is given in Algorithm 1.

**Data:** Population: *n* individuals *Y*_1_, … *Y*_*n*_

**Result:** Partition: *Cluster* vector of size *n* taking values in [1..*k*]

/* Step 0: Initialization                                      */

*k* individuals *C*_1_, *C*_2_, …, *C*_*k*_ are randomly chosen in *Y*_1_, … *Y*_*n*_

*s* ← 0

*Cluster*_0_ ← (0, 0, …, 0) /* vector of size *n*                              */

**repeat**

 *s* ← *s* + 1

 /* Step s.1, phase expectation                                  */

  **for** *i in 1*..*n* **do**

   **for** *j in 1*..*k* **do**

    Compute *DistF*_*i*, *j*_ (The Fréchet distance between *Y*_*i*_ and *C*_*j*_)

    *Clusters*_*S*_(*i*)←*j* such that *DistF*_*i*, *j*_ is smaller than *Dist*_*i, j*′_ for *j*′ ≠ *j*

   **end**

  **end**

  /* Step s.2, phase maximization                                */

  **for** *j in 1*..*k* **do**

   Compute *M*_*j*_, the Fréchet mean of clusters *j* (that is the Fréchet mean of all the *Y*_*i*_ such that *Cluster*_*S*_(*i*) == *j*)

  **end**

**until** *Cluster*_*S*_ == *Cluster*_*S* − 1_ or *s* >*Max_Iteration*

            **Algorithm 1:** kmlShape

### 2.2 Extension to the Fréchet distance

#### 2.2.1 Fréchet distance

The Fréchet distance was introduced by Maurice Fréchet in [14]. Informally, it is often compared to a leash between two trajectories. The Fréchet distance is the minimum length of a leash that would separate a master from his dog walking at different speeds along two trajectories. In other words, each point of each trajectory is associated with the nearest point on the other trajectory. The Fréchet distance is then the longest link between the two trajectories.

Mathematically: let $d((\begin{array}{c} x_{1}\\ y_{1} \end{array}),(\begin{array}{c} x_{2}\\ y_{2} \end{array}))=\sqrt{{\left(x_{1}-x_{2}\right)}^{2}+\left(y_{1}-y_{2}\right){)}^{2}}$ be the Euclidian distance between points $\left(\begin{array}{c} x_{1}\\ y_{1} \end{array}\right)$ and $\left(\begin{array}{c} x_{2}\\ y_{2} \end{array}\right)$. Let *P* and *Q* be two curves from [0, *t*] to R. A reparameterization of the interval [0, *t*] is a continuous function, increasing and surjective from [0, *t*] to [0, *t*]. We denote A the set of all reparameterizations of [0, *t*]. Let *α* and *β* two reparameterizations in A and let *s* be a real belonging to [0, *t*].

Intuitively, curve *P* can be regarded as the mobile trajectory that would travel at constant speed (e.g., two centimeter per second). So *P* ∘ *α* is the same trajectory as *P*, but covered by the mobile with a variable speed, speed defined by *α* (e.g., *α* can set 1 centimeter per second as 1 ≤ *s* ≤ 3 and then 3 centimeters per second as 3 < *s* ≤ *t*).

The distance between curves *P* and *Q* reparameterized by *α* and *β* at time *s* is the distance between $\left(\begin{array}{c} \alpha (s)\\ P(\alpha (s)) \end{array}\right)$ and $\left(\begin{array}{c} \beta (s)\\ Q(\beta (s)) \end{array}\right)$, that is $d_{\alpha ,\beta ,s}\left(P,Q\right)=d((\begin{array}{c} \alpha (s)\\ P(\alpha (s)) \end{array}),(\begin{array}{c} \beta (s)\\ Q(\beta (s)) \end{array}))$. The distance between *P* and *Q* reparameterized by *α* and *β* is the maximum of the distances *d*_*α*, *β*, *s*_(*P*, *Q*) while *s* varies from 0 to *t*: *d*_*α*, *β*_(*P*, *Q*) = *Max*_*s*_(*d*_*α*, *β*, *s*_(*P*, *Q*)). Then the Fréchet distance between *P* and *Q* is the smallest possible maximum between *P* and *Q* after reparameterization of *P* and *Q*: *DistFrechet*(*P*, *Q*) = *d*_*α*, *β*_(*P*, *Q*).

The definition is the same in the discrete case with the exception that *s* takes values between 0 and *t* by intervals. Note that, contrarily to several classical distances, the calculation of Fréchet distance does not require the same number of measurements or the same time points on the two trajectories. Therefore, it can be used to cluster irregular trajectories, the use of imputation methods for longitudinal data [45–48] is not necessary.

From a computational point of view, the Fréchet distance is rather easy to determine [49] but the calculation time is longer than that required for Euclidian distance: *O*(*t*^2^) (the details of all the computational complexity are given in Appendix).

#### The generalized Fréchet distance

**Fig 3:**
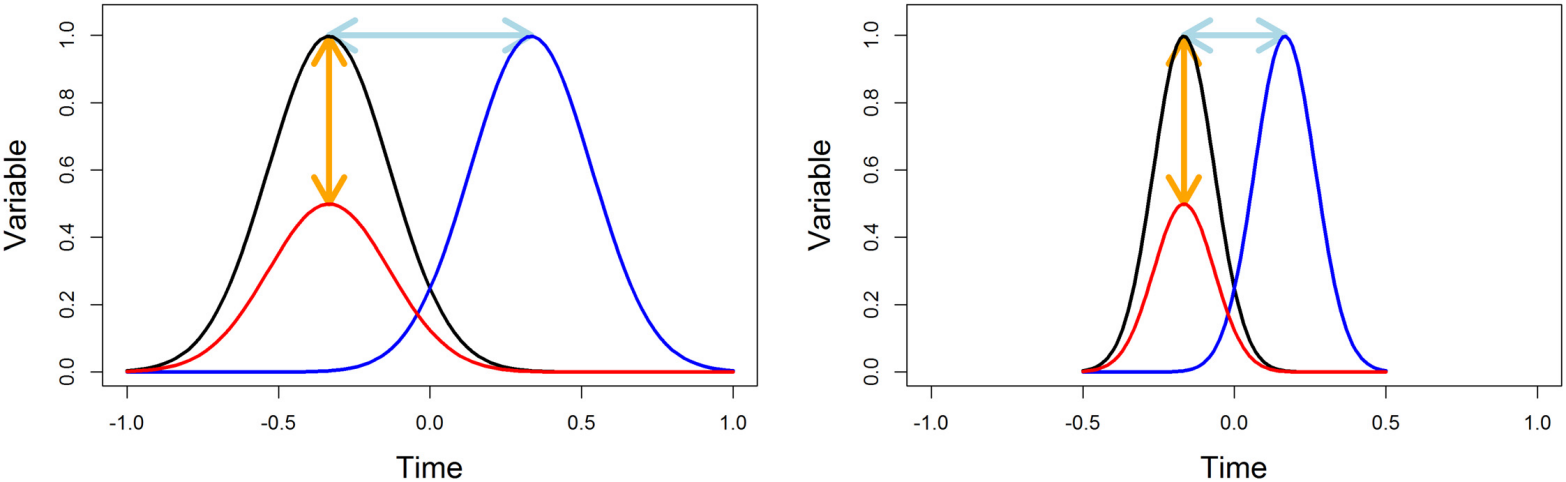
Scale change. (a) *i*_1_ is closer to *i*_2_ than to *i*_3_ (b) *i*_1_ is closer to *i*_3_ than to *i*_2_.

Fréchet has given the seminal definition within the context of two mathematical curves *P* and *Q*. Within the context of real data, there is a relative-scale issue. The variable of interest and the time variable are not measured using the same unit. This can be an important issue since a scale changes impact the Fréchet distance. Fig 3.a shows three trajectories. According to the Fréchet distance, *i*_1_ is closer to *i*_2_ than to *i*_3_ (the segments that materialize the distances between the trajectories are dotted). If the scale of the X-axis is changed (Fig 3.b), *i*_1_ will be closer to *i*_3_ than to *i*_2_.

This scale-change is not trivial because it impacts the partitioning. This lead to the following definition: the generalized Fréchet distance of parameter lambda between two curves *P* and *Q* is the Fréchet distance obtained after an affine transformation $A:\left(\begin{array}{c} x\\ y \end{array}\right)\to \left(\begin{array}{c} \lambda .x\\ y \end{array}\right)$; that is, $DistFrechet_{\lambda }\left(P,Q\right)=Inf_{\left(\alpha ,\beta \right)\in A^{2}}Max\left(P\circ \alpha \circ A,Q\circ \beta oA\right)$. This is what we called *the generalized Fréchet distance*. *λ* is the time-scale parameters.

One should notice that when *λ* = 0, the Fréchet distance matches with the Dynamic Time Warping (DTW) distance (i.e., as in DTW, horizontal shifts have no impacts. See appendix A for more details). On the opposite, when *λ* tends to +∞, then *DistFrechet*_*λ*_ tends toward the classical maximum distance.

Therefore, the generalized Fréchet distance is a generalization of shape-respecting distance (like DTW) but also of other classical distances (Maximum). Herein, for the sake of simplicity, the generalized Fréchet distance will be referred as to the Fréchet distance.

#### The Fréchet mean between two trajectories

As mentioned above, Classification-Expectation-Maximization algorithms require the calculation of a mean. Informally, the Fréchet mean between two trajectories is the middle of the leash that links the dog to the master when each goes along its own way.

More precisely, calculating the Fréchet distance requires the explicit calculation of the two reparameterizations *α* and *β* that minimize *DistFrechet*_*λ*_(*P*, *Q*). Using these two functions, it is obvious to define the Fréchet mean as the mean of the distances between the points of the two trajectories when these trajectories are run at speeds *α* and *β*: $MeanFrechet_{\lambda }(P,Q)=(\left(\begin{array}{c} \frac{P\circ \alpha (\lambda .x(1))+Q\circ \beta (\lambda .x(1))}{2}\\ \frac{P\circ \alpha (y(1))+Q\circ \beta (y(1))}{2} \end{array}\right),\ldots ,\left(\begin{array}{c} \frac{P\circ \alpha (\lambda .x(a))+Q\circ \beta (\lambda .x(a))}{2}\\ \frac{P\circ \alpha (y(a))+Q\circ \beta (y(a))}{2} \end{array}\right))$ that we will write $MeanFrechet_{\lambda }\left(P,Q\right)=(\frac{P\circ \alpha \circ A+Q\circ \beta \circ A}{2})$

**Fig 4:**
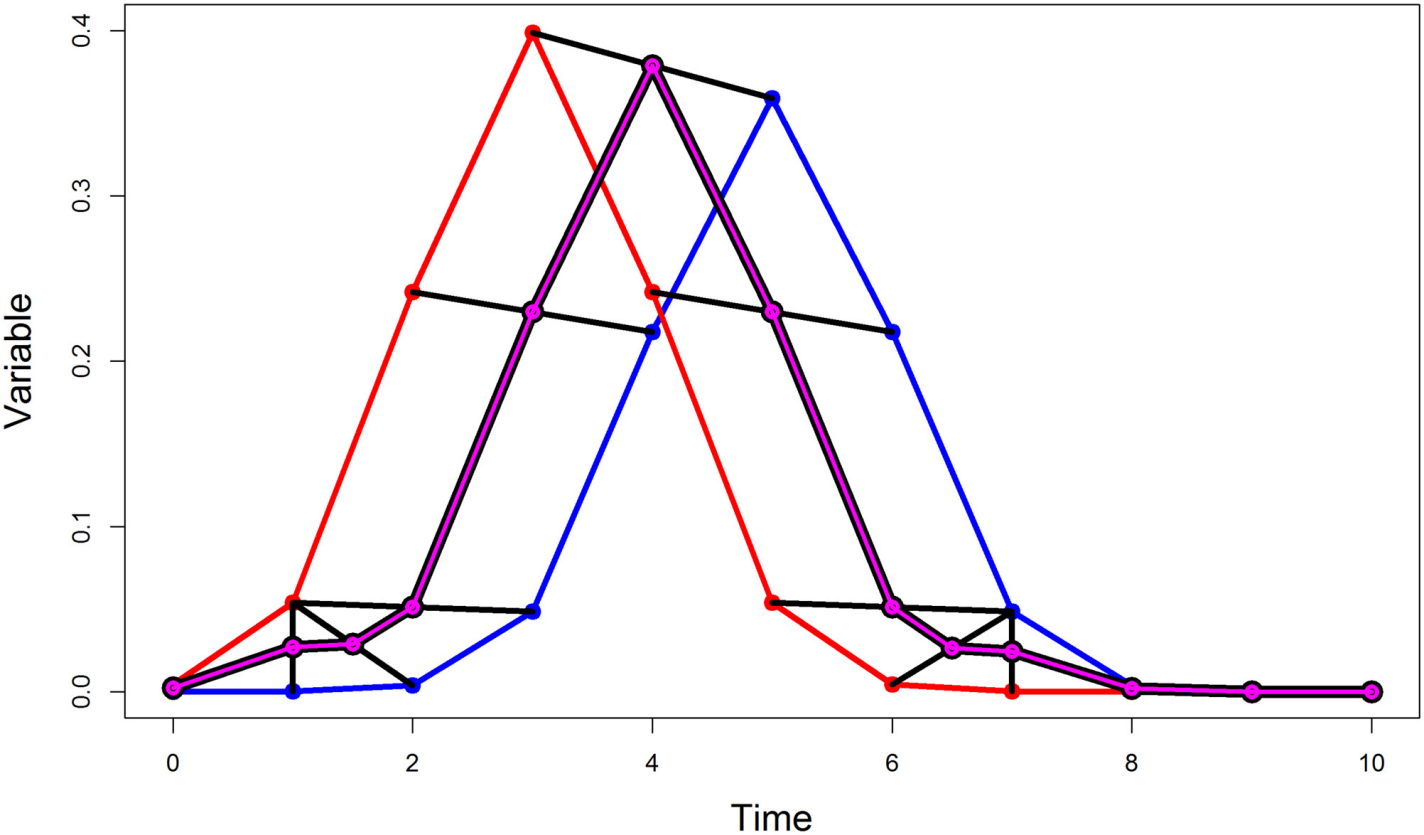
The Fréchet mean. Two trajectories *P* and *Q* are in red and blue, respectively. The segments linking *P* to *Q* after reparameterization are in black. The mean trajectory as defined by the middles of these segments is in violet.

An example of the Fréchet mean is given Fig 4.

The Fréchet mean of the two curves *P* and *Q* weighted by coefficients *p* and *q* works on the same principle with a weighting on each curve: $MeanFrechet_{\lambda }\left(P,p,Q,q\right)=\frac{p.P\circ \alpha \circ A+q.Q\circ \beta \circ A}{p+q}$.

#### Generalization to n curves

The definition of the Fréchet mean may be extended to *n* curves. However, the complexity of the algorithm (*O*(*t*^*n*^)) would not be realistic for the analysis of real data, even of very small size.

However, the Fréchet mean with *n* curves may be approximated with less complexity. The calculation of the Fréchet mean between two curves is reasonable (*O*(*t*^2^)). In a population of *n* individuals, it is possible to combine pairs of individuals (with weight 1), then combine the so-obtained means (weighted by the number of individuals that generated them) until obtaining a unique mean. The calculation cost of this “step by step” mean is *O*(*n*.*t*^2^).

Obviously, the order in which the combinations are made has an impact on the final result. Let us mention three possible variants:

- **RandomAll**: the *n* individuals are randomly scattered on the leaves of a complete binary tree (each knots has either zero or two leaves) having depth *h* with 2^*h*^ ≤ *n* < 2^*h*+1^. The value of each non-terminal leaf is the mean of the two children-leaves. The Fréchet mean is thus the value of the tree root. (Informally, this structure is close to that of a tennis tournament). The complexity of this method is *O*(*nt*^2^).
- **Hierarchical**: the combination order between individuals is fixed in a deterministic way through an ascending hierarchical classification; the closest individuals being combined first. The complexity of this method is *O*(*n*^2^ *t*^2^).
- **RandomSubset**: This method is the RandomAll method applied to a sample of randomly selected individuals. The complexity of the method is *O*(*n*_0_ *t*^2^), *n*_0_ being the size of the random sample.

The means obtained through RandomAll and Hierarchical are very close and, in the case of simulations with artificial data, are also very close to the real mean. The choice of one of theses two methods has thus no impact on the final partitioning. On the contrary, the performance of RandomSubset is dependent on the sample size. Besides, the Hierarchical method is deterministic, which, in the case of an algorithm run several time (such as k-means) is a disadvantage because, in case of convergence toward a local maximum, an additional run of the algorithm will lead to the same maximum. Finally, its complexity is *O*(*n*^2^ *t*^2^) whereas that of randomAll is *O*(*nt*^2^). Thus, it is RandomAll that should be preferred.

### 2.3 Data size reduction

#### Reduction of the number of individuals

**Fig 5:**
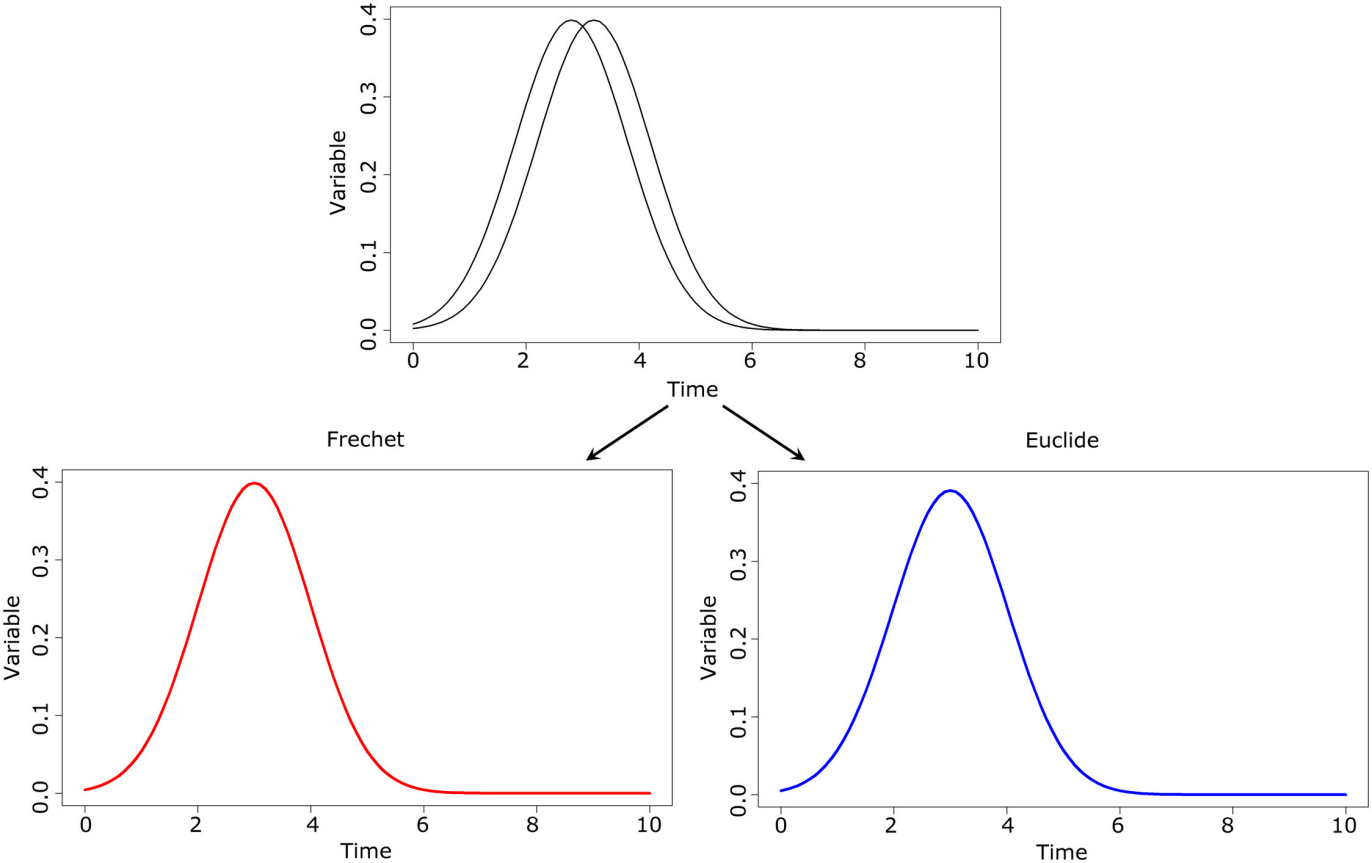
Comparison between Euclidian mean and the Fréchet mean in case of two close curves. The means are almost identical.

The use of the Fréchet mean approximation shifts the complexity of our first algorithm from *O*(*t*^*n*^) to *O*(*nt*^2^). This is an important gain, however insufficient for applying the method to large databases (thousand or tens of thousand individuals). One optimization option is to reduce the number of individuals by an identification of a small number of comparable trajectories. This suggestion of simplification is based on two facts: i) in large populations, some groups of individuals have close trajectories (because the limited number of typical trajectories); this is all the more true as the population becomes larger; ii) when two trajectories are very close, the Euclidian mean and the Fréchet mean are close (see Fig 5). It becomes then locally satisfactory to approximate the Fréchet mean through the Euclidian mean.

With these facts, in case of large populations, it is convenient to replace close groups of individuals by representatives (in the same way senators represent populations of states). In addition, the Fréchet mean may be approximated through the Euclidian mean without changing the forms of the trajectories. In the end, this reduction of the number of individuals may be obtained using a classical classification algorithm such as k-means with a Euclidian distance.

Practically, k-means is carried out on a population of size *n*, with, say, *n*_*S*_ = 128 groups. This is equivalent to make the not very constraining hypothesis that there is a set of 128 representative trajectories so that each individual trajectory is close to at least one of them. The cost of this preliminary classification (we would conveniently name “election”) is *O*(*n*_*S*_ *nt*). Afterwards, kmlShape with weighting may be used with *n*_*S*_ senators stemming from the election. The cost of kmlShape is then *O*(*n*_*S*_ *t*^2^). The overall complexity is *O*(*n*_*S*_ *nt* + *n*_*S*_ *t*^2^).

#### Reduction of the number of measurements

In an orthogonal way, it is generally possible to simplify the trajectories by reducing the number of measurements made without much loss of information. These techniques are known as “Segmentation Time Series” [50, 51], “Line-simplification” [52] or “Trajectories compression” [53] In his survey, Keogh proposes three kinds of methods: the Sliding Windows, the Top-Down, and the Bottom-up. For our purpose, the Top-Down are the ones that have the best complexity. In this article, we will focus on Douglas-Peuker algorithm [54], also known as Ramers algorithm [55] or “Iterative End-Points Fits” [56].

Let us consider a trajectory *Y* of length *t* and an *ϵ*. The Douglas-Peuker algorithm [54] allows finding a curve *Y*^*DP*^ of length *t*_*DP*_ ≤ *t* so that the distance (projection of one point of one curve on the other curve) between *Y* and *Y*^*DP*^ is, in each point, less than *ϵ*. The Douglas-Peuker algorithm is recursive; as long as the simplified trajectory *Y*^*DP*^ is not at a distance less than epsilon from the original trajectory *Y*, the point of *Y* the farthest from *Y*^*DP*^ is added to *Y*^*DP*^. This algorithm makes it possible to set the quality of the approximation of *Y* through *Y*^*DP*^. Note that many amelioration of this algorithm exist [53].

**Fig 6:**
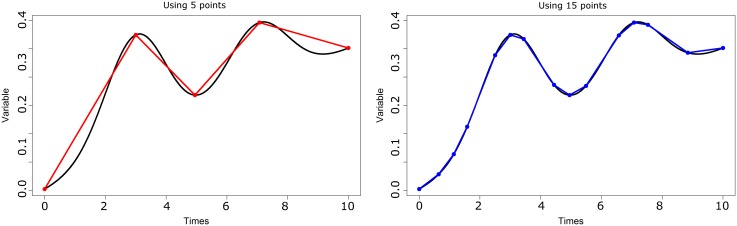
Approximation of a trajectory (in black). (a) using 5 points; (b) using 15 points.

In our present problematic, it may be more interesting to set the adequate length *t*_*DP*_ for the simplified trajectory because this length has a direct impact on the computation time. This may be obtained through a simple modification of Douglas-Peuker algorithm. Instead of considering a calculation-stopping condition that depends on the distance between *Y* and *Y*^*DP*^, we may choose to set the maximum number of points for *Y*^*DP*^: as long as the simplified trajectory *Y*^*DP*^ has less than *t*_*DP*_ points, the point of *Y* the farthest from *Y*^*DP*^ is added to *Y*^*DP*^. With *n*_*S*_ individuals, the complexity of this algorithm is $O(t_{DP}^{2}n_{S}t)$. Fig 6 shows the two types of simplification with 5 and 15 points, respectively.

Note that the modification of the stopping condition induces that the error is no longer explicitly controlled. In some specific cases, this might lead to a simplified trajectory that is no longer close to the initial trajectory (for example, the trajectory sin(*t*) with *t* in [0, 3*π*] approximated with only 3 points). To inform the user on the size of the error, the modified Douglas-Peuker algorithm returns the greatest distance between the simplified curve and the initial curve. Thus, if the user has no direct control on the error, he/she has an estimation of it. He/She can then decide whether the size of the error seems too big for him/her to increase the number of points used by the Douglas-Pecker algorithm. The user may feel also free to use the classical Douglas-Pecker algorithm (control the error but not the number of points). In the latter case, the time complexity of the algorithm kmlShape is not guaranteed.

### 2.4 Overall complexity

In the end, the election cost is *O*(*n*_*S*_ *n*_*t*_). The cost of senator simplification is $O(t_{DP}^{2}n_{S}t)$. One may then use kmlShape with the *n*_*S*_ simplified senators at cost $O(n_{S}t_{DP}^{2})$. The overall complexity is $O(n_{S}nt+t_{DP}^{2}n_{S}t+n_{S}t_{DP}^{2})$, *t*_*DP*_ and *n*_*S*_ being constants set by the user. So, the final complexity is *O*(*nt*).

**Fig 7:**
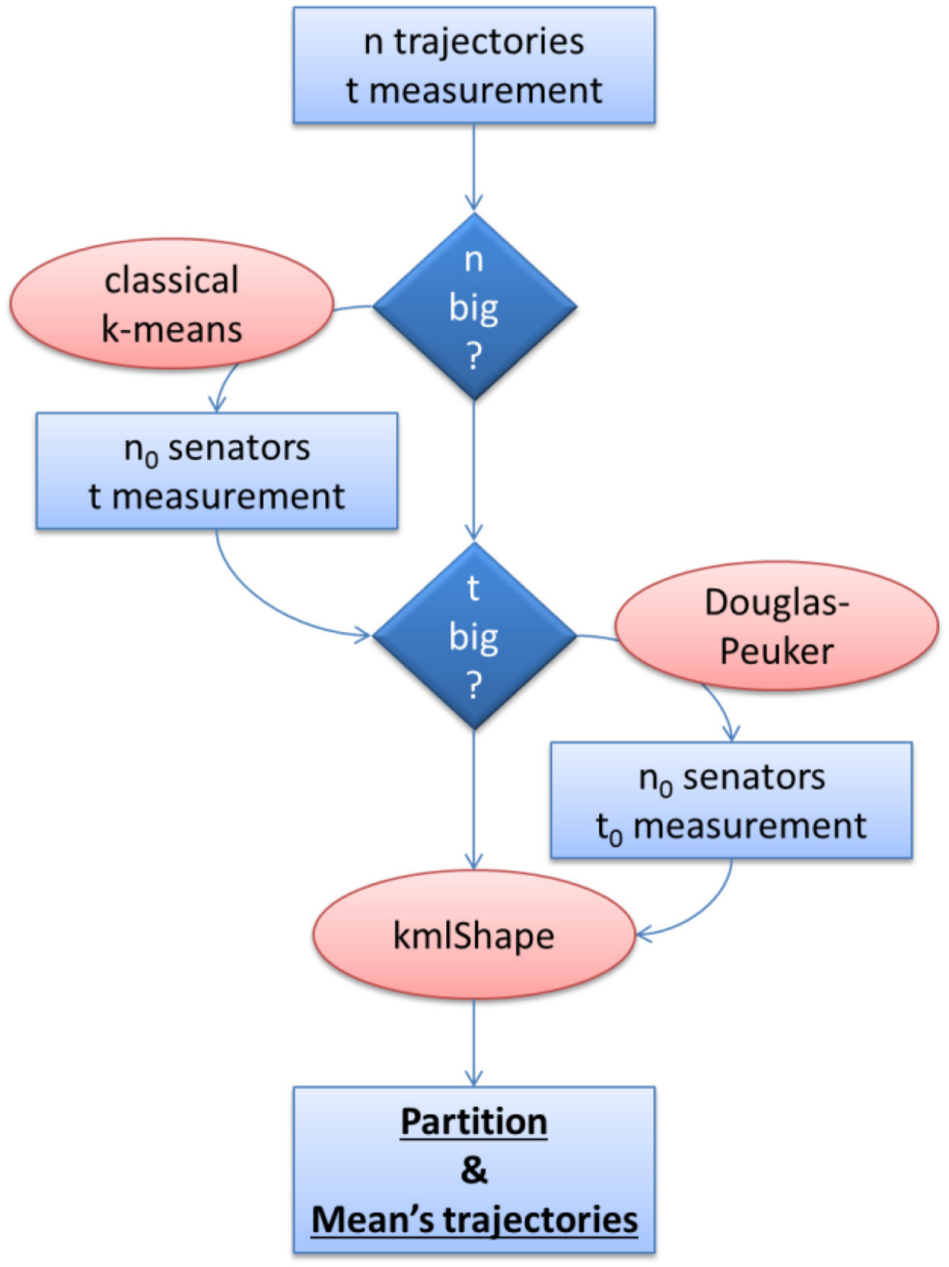
Steps for data partitioning with kmlShape the final complexity is in *O*(*nt*).

Fig 7 summarizes the steps needed to partition data using algorithm kmlShape in a reasonable time.

## 3 Performance assessment

### 3.1 Simulation study

#### Generation of artificial data

**Fig 8:**
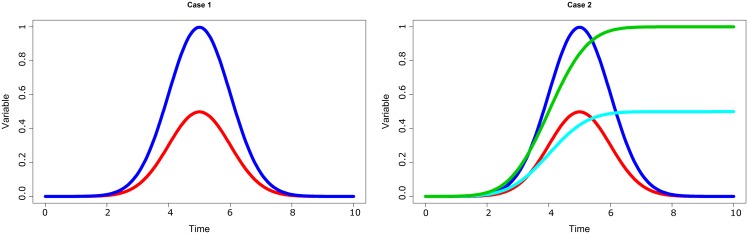
Artificial data. (a) Case 1: *f*_*A*_ is in red, *f*_*B*_ in green; (b) Case 2: *f*_*A*_ is in red, *f*_*B*_ in green, *f*_*C*_ in deep blue and *f*_*D*_ in light blue.

Let us consider three populations of small (*n* = 20, *t* = 21), medium (*n* = 40, *t* = 41), and large size (*n* = 500, *t* = 501). Each population has *k* subgroups. Each subgroup *G* is defined by a typical trajectory *y* = *f*_*G*_(*x*). We have considered two cases:

- **Case 1**: two groups A and B (k = 2) with *f*_*A*_(*x*) = *ψ*(*x*, 0.5, 0.1) × 0.125 and *f*_*B*_(*x*) = *ψ*(*x*, 0.5, 0.1) × 0.25.
- **Case 2**: four groups A, B, C and D (k = 4) with *f*_*A*_(*x*) = *ψ*(*x*, 0.5, 0.1) × 0.125, *f*_*B*_(*x*) = *Φ*(*x*, 0.4, 0.1) × 0.5, *f*_*C*_(*x*) = *ψ*(*x*, 0.5, 0.1) × 0.25 and *f*_*D*_(*x*) = *Φ*(*x*, 0.4, 0.1).

with *ψ* the normal law distribution $\psi \left(x,m,s\right)=\frac{1}{s\sqrt{2\pi }}exp(-\frac{1}{2}{\left(\frac{x-m}{s}\right)}^{2})$ and *Φ* its cumulative distribution function (see Fig 8).

Then each trajectory *y*_*i*_ belonging to subgroup *G* is a distortion of *f*_*G*_. To create *y*_*i*_, we choose a coefficient of distortion *σ*. Three types of distortion may be considered:

- **Simple distortion** it consists in a mere horizontal translation of *f*_*G*_: *y*_*ij*_ = *f*_*G*_(*x*_*ij*_ + *b*_1_), with $b_{1}∼U\left(-\sigma ,+\sigma \right)$
- **Multiple distortion** it consists not only in vertical and horizontal translations but also in vertical and horizontal deformations (compression and stretching): *y*_*ij*_ = *a*_2_.*f*_*G*_(*a*_1_.*x*_*ij*_ + *b*_1_) + *b*_2_, with $\left(a_{1},a_{2}\right)∼U{\left(1-\sigma ,1+\sigma \right)}^{2}$ and $\left(b_{1},b_{2}\right)∼U{\left(-\sigma ,+\sigma \right)}^{2}$.
- **Noisy distortion** it consists in a multiple distortion (as described above) together with a Gaussian random noise: *y*_*ij*_ = *a*_2_.*f*_*G*_(*a*_1_.*x*_*ij*_ + *b*_1_) + *b*_2_ + *e*_*ij*_, with $\left(a_{1},a_{2}\right)∼U{\left(1-\sigma ,1+\sigma \right)}^{2}$, $\left(b_{1},b_{2}\right)∼U{\left(-\sigma ,+\sigma \right)}^{2}$ and $e_{ij}∼N\left(0,\sigma \right)$.

with U(a,b) the uniform distribution with minimum *a* and maximum *b*. For each type of distortion, *σ* takes values in {0.05, 0.1, 0.25}. At the end, (3 possible populations) times (2 possible cases) times (3 possible distortions) times (3 possible *σ*) gives 54 possible datasets. Each dataset was generated 500 times.

Small datasets were partitioned using (1.a) kmlShape (methods randomAll, *λ* = 0.1), (1.b) kmlShape using DTW (methods randomAll, *λ* = 0), (1.d) classical Euclidian k-means, and (1.e) fdakma [27, 31]. Medium datasets were partitioned using (2.a) kmlShape (randomAll, *λ* = 0.1), (2.b) kmlShape using DTW (randomAll, *λ* = 0), (2.c) kmlShape with simplification (*n*_*S*_ = 32, *t*_*DP*_ = 21, randomAll, *λ* = 0.1), (2.d) classical Euclidian k-means and (2.e) fdakma.

Large datasets were partitioned using (3.c) kmlShape with simplification (*n*_*S*_ = 32, *t*_*DP*_ = 21, randomAll, *λ* = 0.1) and (3.d) classical Euclidian k-means; the other methods were too time-consuming.

The study of (1.a, 1.b, 1.d, 1.e, 2.a, 2.b, 2.c, 2.d and 2.e) will allow us to compare kmlShape using Fréchet, kmlShape using DTW, classical k-means and fdakma in various conditions. The comparison of (2.a) and (2.c) will allow us to study the impact of the simplification procedures. (3.c) and (3.d) will allow the comparison of kmlShape with simplification and k-means performance on large data set.

The indicated parameters were chosen because they reflect an equilibrium between a slight deformation of the original data (that requires high *n*_*S*_ and *t*_*DP*_ values) and a reasonable calculation time (that requires low *n*_*S*_ and *t*_*DP*_ values).

### 3.2 Performance

To measure the performance, we used the Correct Classification Rate (cRate) which is the percentage of agreement between the partitioning found *P* and the true partitioning *P*_*T*_. We have also used the adjusted Rand Index (aRand) [57] which is a variant of the Rand Index [58]; the cRand index being the proportion of pairs of individuals (*i*, *j*) who are either in the same cluster in *P* or in *P*_*T*_ or in separate clusters in *P* or in *P*_*T*_. The adjusted rand index is simply aRand = (cRand − theoretical cRand) / (Maximum cRand − theoretical cRand). This adjustment makes the aRand take value 0 when it measures the agreement between two random partitions. These two measures of agreement between classifications have been already used by several authors [5, 9, 30, 59].

## 4 Results

### 4.1 Method comparisons

The respective performances of kmlShape, fdakma, and k-means with small and medium datasets, case 1 and 2, are shown Table 1. We observed that kmlShape performs better than fdakma and k-means regarding the classification indices. The same was found when only one specific subgroup was analyzed (e.g., only Case 1 with *σ* = 0.05). However, the differences between kmlShape and the other methods were more marked in Case 1 than in Case 2. Also, these differences tended to decrease slightly when *σ* increased.

**Table 1:** Performance of classical k-means, fdakma, and kmlShape in case of small and medium datasets (mean value ± standard deviation).

	Classical k-means	kmlShape	fdakma
Case 1			
cRate	0.75 (± 0.12)	0.84 (± 0.15)	0.57 (± 0.07)
aRand	0.56 (± 0.2)	0.71 (± 0.26)	0.37 (± 0.1)
Case 2			
cRate	0.66 (± 0.14)	0.94 (± 0.12)	0.57 (± 0.07)
aRand	0.14 (± 0.27)	0.84 (± 0.32)	0.01 (± 0.06)

Note that for these two examples, the results of kmlShape with *λ* = 0.1 and kmlShape using DTW (i.e. *λ* = 0) were identical. Thus we give only the results of kmlShape with *λ* = 0.1.

### 4.2 Impact of simplification in terms of time or number of individuals

The respective performances of kmlShape, classical k-means, and simplified kmlShape with medium size data are shown Table 2. We observed that the performance of simplified kmlShape was close to (similar or slightly lower) the performance of kmlShape without simplification. In all cases, the performance of kmlShape was clearly better than that of a classical partitioning.

**Table 2:** Performance of classical k-means, kmlShape, and simplified kmlShape with medium datasets (mean value ± standard deviation).

	Classical k-means	kmlShape	Simplified kmlShape
Case 1			
cRate	0.64 (± 0.13)	0.94 (± 0.12)	0.94 (± 0.12)
aRand	0.13 (± 0.25)	0.84 (± 0.31)	0.82 (± 0.32)
Case 2			
cRate	0.75 (± 0.11)	0.84 (± 0.15)	0.82 (± 0.14)
aRand	0.56 (± 0.19)	0.72 (± 0.25)	0.7 (± 0.24)

The performances of classical k-means and simplified kmlShape with large datasets are shown Table 3. The simplified kmlShape outperformed clearly the classical k-means. We also observed that the performance of kmlShape with simplification was quite close to that of kmlShape without simplification.

**Table 3:** Performances of classical k-means and simplified kmlShape with large data (mean value ± standard deviation).

	Classical k-means	Simplified kmlShape
Case 1		
cRate	0.59 (± 0.12)	0.92 (± 0.15)
aRand	0.09 (± 0.22)	0.79 (± 0.38)
Case 2		
cRate	0.71 (± 0.11)	0.8 (± 0.15)
aRand	0.56 (± 0.18)	0.68 (± 0.23)

### 4.3 Application to real data

#### Cohort ICTUS

Ictus (see S1 File) is a cohort of 1380 patients with Alzheimer disease followed-up in 12 European countries [60, 61]. These patients were included between February 2003 and July 2005 in 29 centers specialized in neurology, geriatrics, psychiatry or psycho-geriatrics with a recognized experience in the diagnosis and management of Alzheimer disease. Most of these patients were seen during memory consultations and included consecutively. These patients were examined at six-month intervals over two years. Each examination included (though not exclusively) an Instrumental Activities of Daily Living (IADL) assessment.

**Fig 9:**
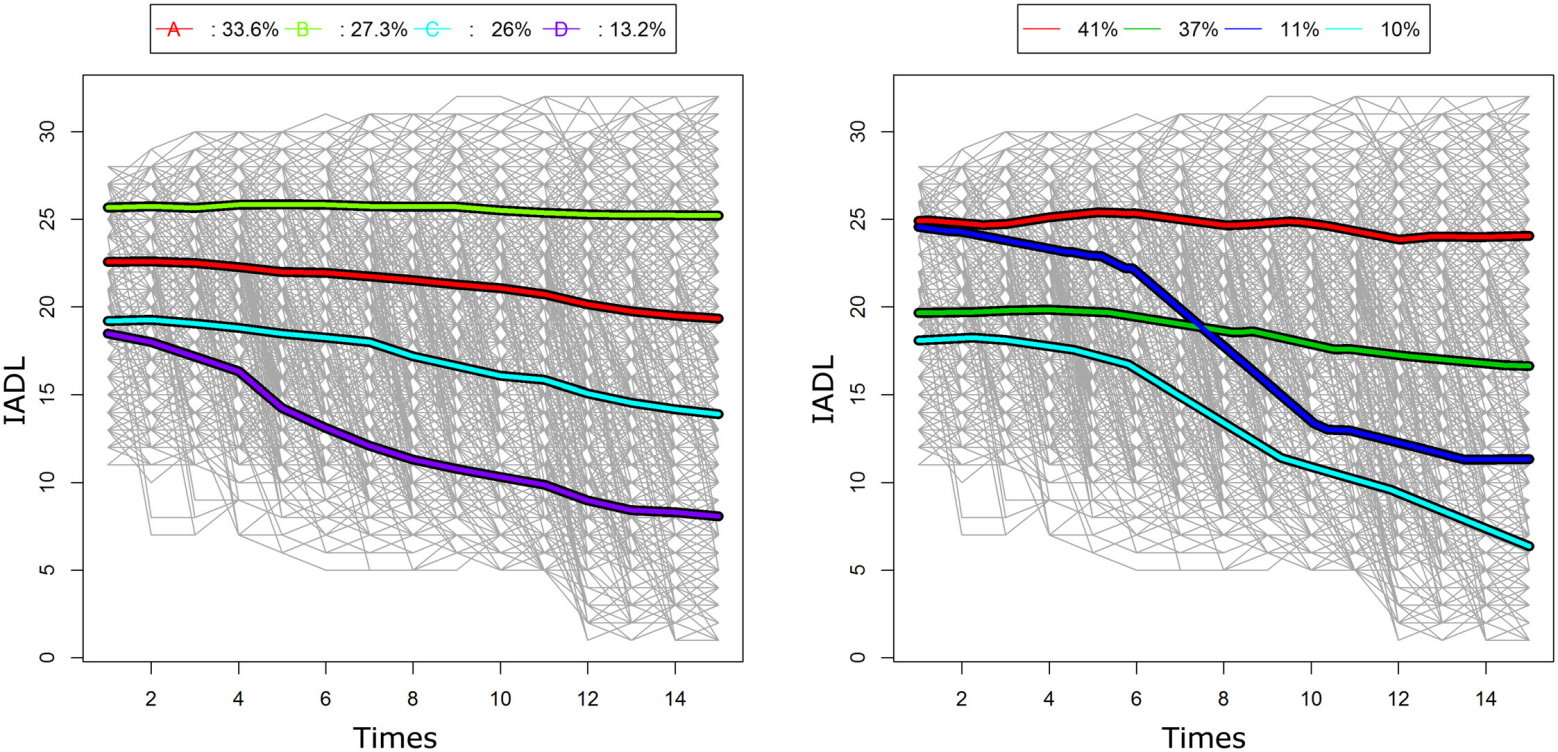
IADL trajectories, in 4 clusters. (a) with a classical method; (b) with kmlShape. kmlShape is able to identify a “rapid decline” cluster that is not be found using the classical method.

A classical analysis of IADL trajectories using either mixture models or k-means revealed 4 groups (Fig 9.a). The main feature of these groups is to show close consistent declines. Using kmlShape (after using the data size reduction *n*_*s*_ = 128, no curve simplification) with 4 groups (Fig 9.b), three of these groups were similar to those found by other classical algorithms whereas a fourth “rapid decline” group was detected by kmlShape only.

The identification of this group makes it possible to predict and anticipate the needs of these patients in terms of informal help or professional care. Such a planning is of utmost importance for the health professionals and the families. Applying the same methods on other functions of these patients (cognition, behavior) would help clarifying the natural history of the disease. Applying these methods before diagnosis would help targeting the population to include in clinical trials for the prevention of Alzheimer disease. Indeed, such trials may use aggressive agents (e.g., monoclonal antibodies) which makes it necessary, from an ethical point of view, to target only the subpopulation with “rapid decline”, which is not possible with the classical classification methods.

#### Quidel database

The QUIDEL database aims to gain better knowledge on hormone profiles of women without fertility problems. This database has been described as the largest existing database on hormone profiles in normally menstruating women and includes ovary ultrasound scans on the day of ovulation [62]. The database includes 107 women and 283 cycles with identification of the day of ovulation and daily titrations of the levels of the four main hormones of the ovulation cycle. It has been already the subject of publications [63, 64]

**Fig 10:**
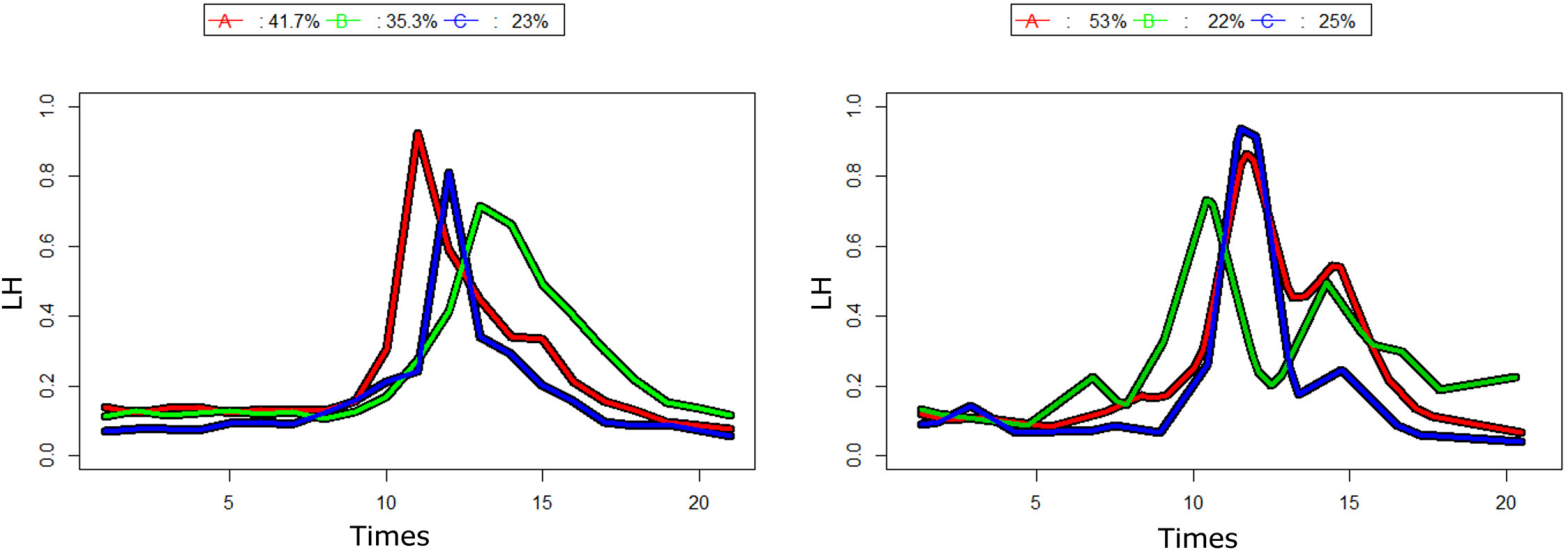
LH trajectories, in 3 clusters. (a) with a classical method; (b) with kmlShape. kmlShape shows typical trajectories with two peaks that are not found with the classical method.

The use of classical classification methods regarding the luteinizing hormone (LH) provided three typical trajectories with similar features but with slight shifts in time (Fig 10.a). This is a typical finding in medicine but is currently strongly questioned [62, 65]. The use of kmlShape (after using the data size reduction *n*_*s*_ = 128, *t*_*DP*_ = 20) with three groups led to identifying: i) a classical profile that would concern 25% of the women; ii) a two LH-peak profile that would concern 22% of the women; and, iii) a profile with a single peak followed by a slow decline over several days that would concern the remaining 53% of the women (Fig 10.b).

These results will enrich the current debate on the role of the LH peak in the maintenance of corpus luteum. Indeed, as indicated by its name, the LH was first described as luteinizing but, as LH peaks occur close to the day of ovulation, LH was made responsible for triggering ovulation. However, recent works [66] have demonstrated that the course of LH during the days that follow ovulation may be important to understand some abnormalities of corpus luteum and, thus, the implantation of the embryo in the uterus. The identification of 22% of women with LH double peak profiles is important for further research in reproductive biology.

#### UCR-CBF

The two last examples (CBF and Trace, see below) are extracted from the “UCR Time Series Classification Archive” [67], a collection of real and artificial datasets dedicated to studies of time series and longitudinal data. Contrarily to the approach we have adopted in our simulation study, these datasets are generated once.

**Fig 11:**
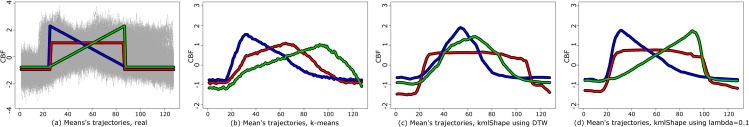
CBF trajectories. (a) real means (b) means found using a classical method; (c) means foung using kmlShape with DTW (d) means found using kmlShape with *λ* = 0.1. kmlShape found the real means.

CBF (Cylinder-Bell-Funnel, [68]) is a dataset of 900 trajectories measured 128 times. These trajectories are divided into three clusters of sizes 302, 300, and 298. The mean trajectories of each cluster are shown Fig 11.a. We partitioned the data with different methods:

- classical Euclidian k-means;
- kmlShape using DWT with simplification (*n*_*S*_ = 64, *t*_*DP*_ = 30, randomAll, *λ* = 0)
- kmlShape with simplification (*n*_*S*_ = 64, *t*_*DP*_ = 30, randomAll, *λ* = 0.1) and

The other methods were too time-consuming or did not converge. The classical classification methods found three groups with identical shapes shifted in time (Fig 11.b). kmlShape using DTW identified three groups with similar shapes but different heights (Fig 11.c). With these two classification methods, the number of misclassified trajectories was quite important (Table 4) and the average trajectories obtained were quite different from the average trajectories used to generate groups (Fig 11.a). kmlShape with *λ* = 0.1 gave good results in terms of individual ranking as in terms of identification of the average trajectory (Fig 11.d).

**Table 4:** Performance of classical k-means, simplified kmlShape using DTW and simplified kmlShape with *λ* = 1 on CBF dataset.

	Classical k-means	Simplified kmlShape with DTW	Simplified kmlShape with *λ* = 1
cRate	0.65	0.59	0.95
aRand	0.34	0.25	0.86

Note that, in this example, the use of the DTW method gave a different (worse) result than the Fréchet mean with *λ* = 0.1. The reasons for this will be discussed in the appendix A.

#### UCR-Trace

**Fig 12:**
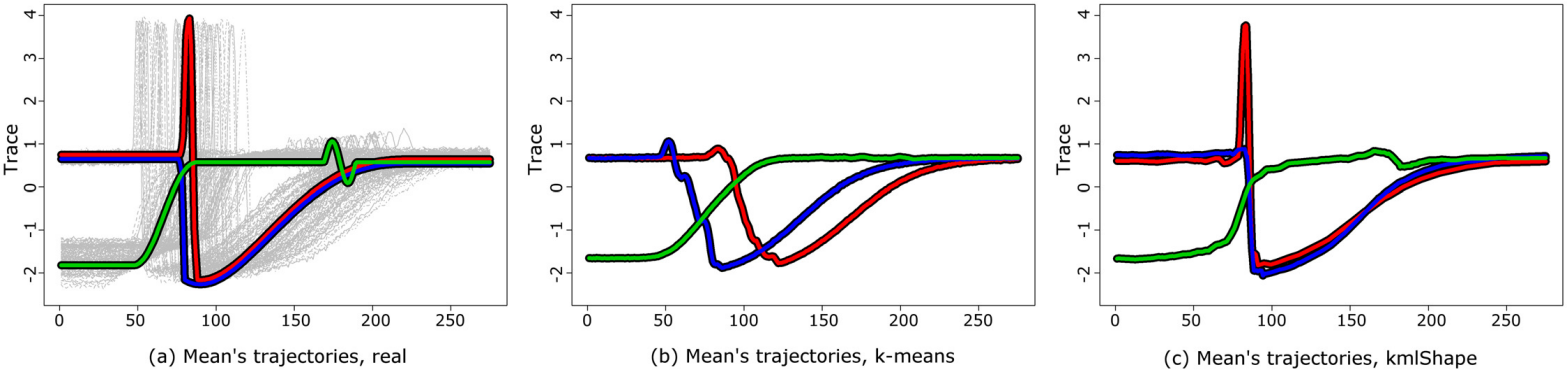
Trace trajectories. (a) real means (b) means found using a classical method; (c) means found using kmlShape with *λ* = 0.1. kmlShape found the real means.

The Trace database [69] (also extracted from the “UCR Time Series Classification Archive”) was obtained from EDF (Electricité de France). The database includes 200 different transient classes (Fig 12.a). We clustered the data using:

- classical Euclidian k-means;
- kmlShape using DWT with simplification (*n*_*S*_ = 64, *t*_*DP*_ = 30, randomAll, *λ* = 0)
- kmlShape with simplification (*n*_*S*_ = 64, *t*_*DP*_ = 30, randomAll, *λ* = 0.1) and

The other methods were too time-consuming or did not converge. kmlShape *λ* = 0.1 and kmlShape using DTW gave exactly the same results (we report here only kmlShape with *λ* = 0.1). The classical classification method identified perfectly one of the three groups (the group in blue Fig 12.b) but failed to distinguish between the two others. The kmlShape identified three groups without errors and found the right average trajectories (Fig 12.c and Table 5.)

**Table 5:** Performance of classical k-means, simplified kmlShape using DTW and simplified kmlShape with *λ* = 0.1 on Trace dataset.

	Classical k-means	Simplified kmlShape
cRate	0.77	1
eRand	0.73	1

## 5 Discussion

In the present article, we introduce kmlShape, a novel method of data partitioning. This method provides clusters on the basis of trajectory shape. This allows especially grouping individuals whose trajectories have similar forms but shifted positions in time. Given the high algorithmic complexity of kmlShape, we present two data-simplification methods that allow reducing the lengths of the trajectories or the number of the individuals of the population under study.

In comparison with other shape-based partitioning methods, kmlShape demonstrated higher performances with the datasets tested whatever the variance, the population size, or the number of clusters. In addition, the search for loss of information due to data simplification has shown that the final partitioning is only slightly affected by this simplification (Table 2). Another advantage was that each of the partition steps (reduction of the number of measurements or of the population size) is mathematically simple, graphically displayable, and easily checkable. At any moment, the user can decide to invalidate any excessive “data simplification”. Finally, with real datasets, kmlShape makes it possible to detect groups of individuals of non-negligible sizes that would not be detected by other classical methods. Thus, the method paves the way to new perspectives in terms of data analysis.

Within the general context of data partitioning, the problem of the optimal number of clusters is still an open issue. Numerous criteria exist, either parametric (BIC [70], AIC [71], AICc [72], global posterior probability [73], …) or non-parametric (Caliskin & Harabatz [74], Ray & Turi [75], Davies & Bouldin [76], …). These criteria are regularly compared using artificial data [77, 78]. With real data, they often suffer from bias. For example, Calinski & Harbatz criteria (which is the best criterion according to both [77] and [78]) often select the lowest number of clusters. Also, different authors advise to choose the number of clusters on the basis of clinical relevance rather than an index [13].

In the case of partitioning using the Fréchet distance, the problem is more complicated because the classical criteria are designed to be used with classical distances. To date, there is no quality criterion that can help selecting the correct number of clusters within the context of respecting-shape partitioning. Finding such a quality index would be a non-negligible progress in the field of data partitioning.

Regarding the choices of *n*_*S*_ and *t*_*DP*_, the present study showed that *n*_*S*_ = 32 and *t*_*DP*_ = 20 is a good compromise between a reasonable simplification and an acceptable calculation time. Obviously, these parameters may be adapted according to the type of data (with complex and long curves, *t*_*DP*_ = 20 seems to be insufficient; with simple curves *t*_*DP*_ = 10 may be sufficient) and the power of the computer involved. In a medium term, new high-performance statistical software programs will probably overcome the current limitations.

The choice of *λ* is more complex. As shown Fig 3, it changes the relative weight of the distance between two trajectories according to the x-axis and the y-axis. If the x-scale and the y-scale are identical, setting *λ* = 0.1 gives ten times more weight to a vertical offset than to a horizontal offset. This case is close to the one shown in the right panel Fig 3: *i*_1_ is close to *i*_3_ because the “horizontal offset” is very important. When *λ* is 1, the horizontal and the vertical offsets have the same importance. When *λ* = +∞, the horizontal offsets becomes very expensive, the Fréchet distance is then identical to the classical maximum distance. When *λ* = 0, the horizontal offsets becomes free, the Fréchet distance is then identical to the dynamic time warping distance. With our artificial examples, a value of *λ* = 0.1 or less allowed a correct identification of the groups. More detail about *λ* can be find appendix A.

When the scales are not the same (which is true in the majority of cases in the present study because one axis represents time and the other the variable of interest), the data can be standardized by dividing by the range of x and multiplying by the range of y. On our real examples, the value used was $\lambda =\frac{Max\left(y_{it}\right)-Min\left(y_{ij}\right)}{Max(j)-Min(j)}\times 0.1$. This value is the one that gave the most relevant results from the clinical point of view and has identified groups undetected by conventional techniques.

## A How to choose *λ*?

The choice of *λ* is of considerable impact on the final clustering. There is no “best” value for *λ* as there is no “best clustering techniques ever”, it must be selected depending on the problem. For that, according to the specific problem, the user has to define the curves that should be considered as “close curves”. To make this decision, it is important to keep in mind the principle of working of *λ*.

**Fig 13:**
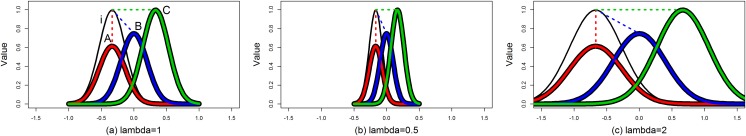
The impact of *λ* on the distance between the trajectory *i* and the clusters mean’s trajectories *A*, *B* and *C*.

Consider Fig 13.a. The black trajectory represents an individual *i*. The three colored trajectories represent three cluster centers. The question is to decide which *i* should be the closest. Suppose that the trajectories represent the intensity of a disease. From a public health perspective, it is important to know when the vaccines should be ready so it is interesting to group *i* with A. For a researcher who wants to understand the disease, the type of disease progression is more important than the time of its outbreak so it will be more relevant to group *i* and *C* because. In some other problems, it might be interesting to group *i* and *B*.

Consider now the Fréchet distance between *i* and the cluster centers (represented by the segments between the curves in dash). In this example, *i* is close to the average trajectory *B* (the dashed blue line is shortest than the green or the red lines). If we represent the same data but divide the scale of the *x* axis by two (Fig 13.b), *i* is closer to *C* (the green dashed line is the shortest). If we represent the same data but multiply the scale of the x by 2 (Fig 13.c), *i* is close to *A* (the red dashed line is the shortest).

From a mathematical perspective, this is easy to understand: the calculation of the length of a segment involves two components; the differences in values along axis *x* and along axis *y*. Changing the scale of *x* changes the relative importance of the two differences (reducing the *x*’ scale decreases the importance of the difference along the axis of *x*). In Fig 13.a, a horizontal shift has a great impact on the calculation of the distance. So *i* is close to *A*. In Fig 13.b, a horizontal shift has a little impact on the distance calculation. So *i* is close to *C*. In extreme cases, when *λ* tends to +∞, a small difference on the x-axis increases greatly the distance between the curves. The Fréchet distance is reached when “man and dog” keep the same abscissa at any point (as a difference of *δ* causes an increase in the distance of *δ* × *λ* which tends to +∞ as *λ* tends to +∞). The Fréchet distance is then identical to the max distance.

Conversely, when *λ* is 0, the distance between points (*x*_1_, *y*_1_) and (*x*_2_, *y*_2_) is just |*y*_1_ − *y*_2_|. The difference along the x-axis has no impact on the distance between the curves. The Fréchet distance matches the DTW.

In summary, the role of *λ* is to allow the user to choose the case in which he wishes to be. Suppose that the initial population is shown Fig 13.b. If, according to the problem, it is relevant to cluster *i* with *A*, then *λ* should be small (*λ* < <1). If *i* should be clustered with *B*, then *λ* = 1 will be a correct choice. If *i* should be close to *C*, then *λ* should be big (*λ* > >1).

## B Appendix: Computational complexities

### B.1 Euclidean distance

The formula for calculating the Euclidean distance is $Dist\left(y_{i.},y_{i^{\prime }.}\right)=\sum_{j=1}^{t}{\left(y_{ij}-y_{i^{\prime }j}\right)}^{2}$, that is subtraction and a square for each *j*, then *t* − 1 additions. The overall complexity is *O*(*t*).

### B.2 The Fréchet distance

The calculation of the Fréchet distance needs the calculation of the distance matrix between each pair of points $\left((\begin{array}{c} j\\ y_{ij} \end{array}),(\begin{array}{c} j^{\prime }\\ y_{i^{\prime },j^{\prime }} \end{array})\right)$. This is a matrix of size *t*^2^. The complexity of the calculation of the distance between each pair of points is a constant, so the complexity of the Fréchet distance is *O*(*t*^2^).

The computing complexity of the Fréchet path is identical because, in addition to the computation of the matrix distance between each pair of points, it only requires browsing the matrix once to find the path.

### B.3 The Fréchet mean between two trajectories

The computing complexity of the Fréchet mean between two trajectories needs the calculation of the Fréchet path (cost: *O*(*t*^2^)). The length of the Fréchet path is bounded by 2*t* − 2. Then the computation of the mean needs up to 2*t* − 2 additions and divisions. Thus the overall complexity is *O*(*t*^2^).

### B.4 The Fréchet mean between two trajectories

The generalization of the Fréchet mean to *n* trajectories requires the calculation of an index for each tuple $\left((\begin{array}{c} j_{1}\\ y_{i_{1}j_{1}} \end{array}),(\begin{array}{c} j_{2}\\ y_{i_{2},j_{2}} \end{array}),...,,(\begin{array}{c} j_{n}\\ y_{i_{n},j_{n}} \end{array})\right)$, that is, a matrix of size *t*^*n*^. The complexity of the calculation is therefore at least *O*(*t*^*n*^).

### B.5 The Fréchet mean, method RandomAll

Method RandomAll merges *n* individuals two by two. It takes *n* − 1 merges (cost: *O*(*t*^2^)). The overall complexity is *O*(*nt*^2^).

### B.6 The Fréchet mean, method Hierarchical

Method Hierarchical computes the Fréchet distance (cost: *O*(*t*^2^)) between all possible couples (*n*(*n* − 1)/2 couples) for a total cost of *O*(*n*^2^ *t*^2^). Then, it merges the *n* individuals two by two. Each merging has a cost of *O*(*t*^2^). The final complexity is *O*(*n*^2^ *t*^2^).

### B.7 The Fréchet mean, method RandomSubset

Method RandomAll merges *n*_0_ individuals two by two. It takes *n*_0_ − 1 merges. Each merge costs *O*(*t*^2^). The overall complexity is *O*(*n*_0_ *t*^2^).

### B.8 k-means

At each stage of k-means, *nk* Euclidean distances (cost: *O*(*t*)) between *n* individuals and *k* groups centers are calculated (cost: *O*(*tnk*)). Then, in each group *g*, the mean of the *n*_*g*_ individuals belonging to the group is calculated; that is, *tn*_*g*_ additions per group with *n* = ∑*n*_*g*_. The final complexity is *O*(*tnk*) (the number of iterations is neglected here because it is generally bounded, it is therefore a constant).

### B.9 kmlShape

At each stage of k-means, *nk* Fréchet distances (complexity *O*(*t*^2^)) between *n* individuals and *k* groups centers are calculated (cost: *O*(*t*^2^ *nk*)). Then, in each group *g*, the mean of *n*_*g*_ individuals in the group is calculated. The cost is *O*(*n*_*g*_ *t*^2^) per group with method RandomAll, $O(n_{g}^{2}t^{2})$ with method hierarchical. The final complexity is *O*(*knt*^2^) with method RandomAll and *O*(*n*^2^ *t*^2^) with method Hierarchical.

### B.10 Douglas-Peuker algorithm

For a curve which must be simplified into *t*_0_ points, each iteration requires the calculation of the distance between the *t* points and the current curve (cost: *O*(*t*.*t*_0_)). This has to be done *t*_0_ times. So, for *n* curves, the complexity is $O(ntt_{0}^{2})$.

## Supporting Information

S1 File A subset of the longitudinal study ICTUS. (CSV)
